# A double-edged sword role of IFN-γ-producing iNKT cells in sepsis: Persistent suppression of Treg cell formation in an Nr4a1-dependent manner

**DOI:** 10.1016/j.isci.2024.111462

**Published:** 2024-11-23

**Authors:** Yingyu Qin, Yilin Qian, Shengqiu Liu, Rong Chen

**Affiliations:** 1 Department of Pathogenic Biology and Immunology, Jiangsu Provincial Key Laboratory of Critical Care Medicine, School of Medicine, Southeast University, Nanjing, Jiangsu, China; 2 Department of Pathogenic Biology and Immunology, School of Medicine, Southeast University, Nanjing, Jiangsu, China; 3 The Affiliated Zhongda Hospital, Clinical Medical College, Southeast University, Nanjing, Jiangsu, China

**Keywords:** Biological sciences, Immune response, Immune system, Immunology, Natural sciences

## Abstract

Sepsis, a leading cause of mortality in intensive care units worldwide, lacks effective treatments for advanced-stage sepsis. Therefore, understanding the underlying mechanisms of this disease is crucial. This study reveals that invariant natural killer T (iNKT) cells have an opposing role in the progression of sepsis by suppressing regulatory T (Treg) cell differentiation and function. The activation of iNKT cells by α-Galcer enhances interferon (IFN)-γ production. Blocking antibodies or transferring IFN-γ-deficient iNKT cells demonstrates that iNKT cells inhibit Treg differentiation through IFN-γ production. Additionally, iNKT cell-mediated Treg inhibition prevents secondary infection caused by *Listeria monocytogenes* during the post-septic phase. The transcriptomic analysis of Treg cells further reveals that the suppressive function of Tregs is impaired by iNKT cells. Finally, we demonstrate that iNKT cells inhibit Treg differentiation in an Nr4a1-dependent manner. Our data uncover the dual function of iNKT cells in sepsis progression and provide a potential treatment target for this adverse long-term outcome induced by sepsis.

## Highlights

• iNKT cell activation worsens early sepsis by inhibiting Treg differentiation
• Long-term Treg inhibition alleviates immunosuppression in the post-septic phase
• iNKT cell-derived IFN-γ suppresses Treg differentiation through inhibition of Nr4a1

## Graphical abstract

**Figure:**
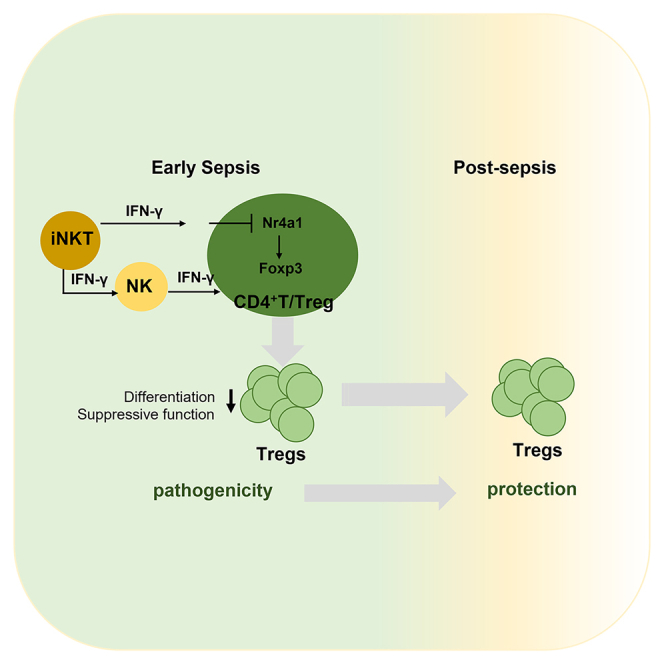

## Introduction

Sepsis is a commonly encountered life-threatening syndrome characterized by an aberrant host response to severe infection, resulting in hyperinflammation and an early onset of cytokine storm that ultimately leads to multiple organ dysfunction.^1^^,^^2^ Following acute sepsis, patients enter a long period of immunosuppression, in which patients succumb to the increased susceptibility to secondary or opportunistic infections.^3^ Advances in understanding the pathophysiology of sepsis have promoted the development of targeted therapies aimed at restoring immune system equilibrium and enhancing patient outcomes, which highlights the necessity for continued research to better understand how to challenge this complex disease.

Natural killer T (NKT) cells are innate-like T lymphocytes that share surface markers and functional characteristics with both natural killer (NK) cells and T cells.^4^ Invariant NKT (iNKT) cells are a subset of NKT cells that express an invariant T cell receptor α chain (TCRα-Vα24–Jα18 in humans and Vα14–Jα18 in mice). Unlike conventional T cells responding to peptide antigens, iNKT cells are lipid-reactive T cells that recognize endogenous or microbial glycolipid antigens presented by CD1d, a major histocompatibility complex class I-like molecule expressed on antigen-presenting cells.^5^ Shortly upon activation, iNKT cells can rapidly secrete abundant amounts of cytokines, predominantly interferon (IFN)-γ and interleukin (IL)-4, which allows them to activate or regulate other immune cells, such as dendritic cells, B cells, NK cells, CD8^+^T cells, and CD4^+^T help cells, through cytokine stimulation or cognate interaction.^6^ Therefore, iNKT cells serve as cellular adjuvants that modulate various immune responses including boosting antimicrobial responses during infection.^7^ A growing body of research has revealed that iNKT cells, serving as the primary instigators of immune response, play pivotal roles in the pathogenesis of sepsis.^8^^,^^9^^,^^10^ In sepsis, the systemic exposure to pathogenic microbial antigens (peptides and lipids) triggers a complex and dysregulated immune response. The initial hyperreactive phase of sepsis is attributed to a substantial release of pro-inflammatory cytokines, including tumor necrosis factor (TNF), IL-1, and IFN-γ, which are secreted by highly activated monocytes, macrophages, and other immune cells.^2^ Therefore, iNKT cells, known for a potent inducer of IFN-γ and other pro-inflammatory mediators, have been considered significant contributors to the dysregulated septic response. Except for promotion of early pathogenesis of sepsis, a recent study has revealed a novel role for iNKT cells, wherein the heightened production of IFN-γ driven by iNKT cells contributes to an augmentation of immunosuppression in post-sepsis.^11^ Therefore, iNKT cells appear to play a long-lasting role in the pathogenesis of sepsis, and the mechanisms underlying the roles of iNKT cells remain elusive. As iNKT cells are potent producers of IFN-γ and can initiate the broader immune response by recruiting and activating other subsets of leukocytes, we thereby hypothesized that iNKT cells regulate other immune cells via IFN-γ to modulate immune response at different stages of sepsis.

Regulatory T (Treg) cells are a subset of CD4^+^T lymphocytes with the properties of negative regulation of immune response to prevent autoimmunity and exaggerated responses to infections.^12^ The rapid increase of Treg cells in severe sepsis and septic shock is well-documented, as they play a crucial role in controlling the initial intense systemic inflammatory response. However, this subsequent negative feedback of the anti-inflammatory process contributes to a long-term inhibitory response that diminishes host resistance to secondary infections, thereby exerting a detrimental effect on patient outcomes.^13^ Several studies have suggested that iNKT cells play diverse roles in modulating peripheral Treg cell differentiation across various experimental settings and disease models.^14^^,^^15^^,^^16^^,^^17^ α-galactosylceramide (α-Galcer), a derivative from a bacterium on the Agelas mauritianus marine sponge, can specifically and strongly activate both human and mouse iNKT cells. Thus α-Galcer has been extensively used as a model antigen for iNKT cell studies. The activation of iNKT cells by α-Galcer has been demonstrated to effectively control autoimmune disorders, such as type 1 diabetes and myasthenia gravis, through the induction of Treg cells.^18^^,^^19^ Ronet et al. reported that activation of iNKT cells can enhance Treg cells to control the inflammatory intestinal disorder.^20^ In contrast, activation of iNKT cells induces rapid pregnancy loss through decrease of Treg cells.^21^ Therefore, we hypothesized that the Treg cells are involved in the pathogenesis of sepsis mediated by iNKT cells, thereby exerting a prolonged impact on the course of sepsis.

In this study, we first determined that α-Galcer-mediated iNKT cell activation enhances sepsis-induced lethality. Activation of iNKT cells by α-Galcer leads to an upregulation of IFN-γ production and a concomitant downregulation of IL-4 secretion in Cecal ligation and puncture (CLP) -induced sepsis. We found that the activation of iNKT cells hampers the differentiation of Treg cells during early sepsis, which serves as an exacerbating factor for iNKT cell-mediated sepsis-induced mortality. Moreover, this inhibitory effect persists for long term in post-sepsis, and transcriptomic analysis of bulk Treg cells revealed that iNKT cells impair the suppressive function of Treg cells, suggesting that iNKT cell-mediated Treg cell inhibition may alleviate immunosuppression in late sepsis. Finally, we determined that IFN-γ-produced iNKT cell inhibits Treg cell differentiation through an IFN-γ-Nr4a1-dependent manner. Our data, therefore, uncover a function of iNKT cells in the progress of sepsis and identify a target for potential treatment of this adverse long-term outcome induced by sepsis.

## Results

### α-Galcer-primed iNKT cells skewing toward Th1 type contribute to exacerbation of sepsis

**Figure 1:**
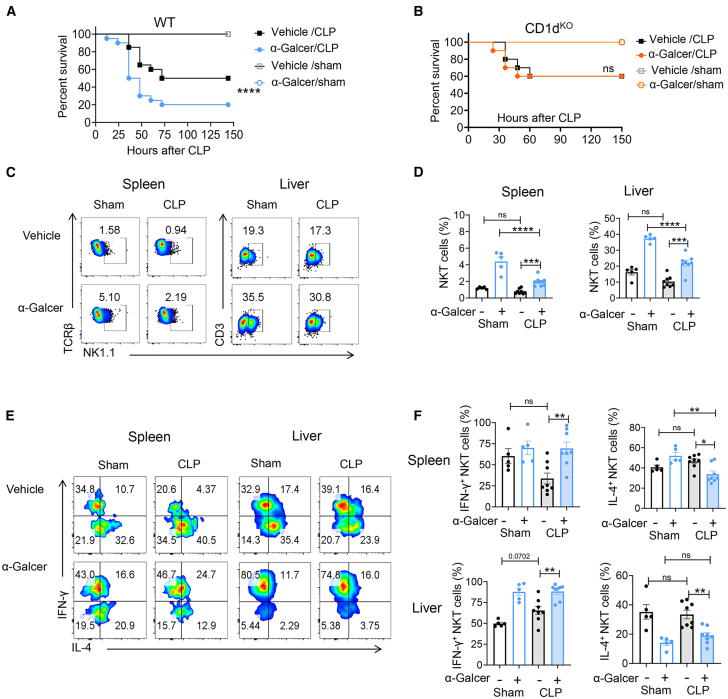
α-Galcer induces Th1-type iNKT cell polarization in sepsis (A and B) The WT mice (A) or CD1d^KO^ mice (B) were intraperitoneally injected with 2 μg α-Galcer in PBS or only PBS (vehicle), followed by CLP or sham surgery. The survival rates of septic mice were assessed over a 150-h monitoring period. WT sepsis group: CLP *n* = 7, Sham *n* = 5; CD1d^KO^ sepsis group: CLP group *n* = 7, sham *n* = 5. Data are representative of two independent experiments. (C–F) The WT mice were intraperitoneally injected with 2 μg α-Galcer in PBS or only PBS (vehicle), followed by CLP or sham surgery. After 12 h of sepsis induction, the frequencies of iNKT cells were examined using TCRβ-APC, CD3-PE, and NK1.1-fluorescein isothiocyanate (FITC) (C and D). The expression levels of IFN-γ and IL-4 in iNKT cells from spleen and liver lymphocytes were analyzed using intracellular fluorescence-activated cell sorting (FACS) analysis (E and F). Each dot represents a mouse sample. Vehicle-sham (*n* = 5), α-Galcer-sham (*n* = 5), vehicle-CLP (*n* = 8), α-Galcer-CLP (*n* = 8). The results are pooled from three independent experiments (C–F). Statistical significance was determined using one-way ANOVA with Tukey tests for multiple-group comparisons; Survival curve comparison was analyzed by log rank test. Data are shown as the mean ± SEM. ∗*p* < 0.05, ∗∗*p* < 0.01, ∗∗∗*p* < 0.001, ∗∗∗∗*p* < 0.0001.

To investigate the roles of iNKT cells in sepsis, we first determined whether activation of iNKT cells affect the survival of CLP-induced sepsis. The α-Galcer treatment resulted in increased mortality in wild-type (WT) mice during sepsis (Figure 1A). However, this effect was abrogated in CD1d knockout mice (CD1d^KO^, defects of CD1d restrict NKT cells), suggesting that activation of iNKT cell contributes to the exacerbation of sepsis (Figure 1B). The frequencies of NKT cells in the spleen and liver were subsequently assessed using flow cytometry following α-Galcer treatment (Figures 1C and 1D). The gating strategy was displayed in Figure S1. α-Galcer significantly increased the frequencies of NKT cells in both sham and sepsis model, which indicates the augmented cell population represents α-Galcer-specific iNKT cells. In addition, the frequencies of iNKT cells were found to be significantly elevated following α-Galcer stimulation in sepsis models compared to non-α-Galcer-treated sepsis models. IFN-γ and IL-4 are two representative cytokines of iNKT cells, and their expression level can indicate the type of iNKT cell to skew toward. Sepsis did not markedly alter IFN-γ and IL-4 production by iNKT cells without α-Galcer treatment. However, upon stimulation with α-Galcer, a significant enhancement of IFN-γ production was observed in iNKT cells, accompanied by a reduction in IL-4 levels (Figures 1E and 1F), indicating a Th1-skewed pro-inflammatory phenotype of iNKT cells.

### Activation of iNKT cells inhibits Treg cell formation during sepsis

**Figure 2:**
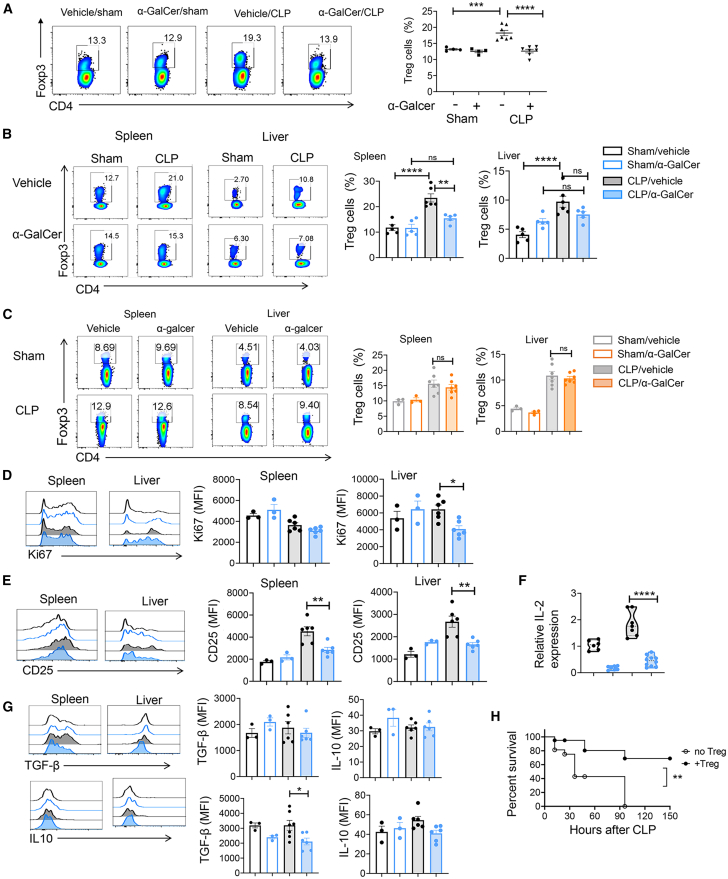
α-Galcer-primed iNKT cells inhibit Treg cell differentiation for long term during sepsis (A) The WT mice were intraperitoneally injected with 2 μg α-Galcer in PBS or only PBS (vehicle), followed by CLP or sham surgery. After 14–18 days of CLP, the frequencies of Treg cells in the spleen of surviving WT mice were assessed by FACS analysis using TCR-APC.Cy7, CD4-FITC, and Foxp3-APC antibodies. The septic surviving mice were obtained from two separate experiments. Sham/vehicle (*n* = 4), Sham/α-Galcer (*n* = 4), CLP/vehicle (*n* = 7), CLP/α-Galcer (*n* = 7). (B and C) The WT mice (B) or CD1d^KO^ mice (C) were intraperitoneally injected with 2 μg α-Galcer in PBS or only PBS (vehicle), followed by CLP or sham surgery. After 48 h of sepsis induction, the frequencies of Treg cells in the spleen and liver were examined. The results are pooled from two independent experiments. WT: Sham/vehicle (*n* = 5), Sham/α-Galcer (*n* = 5), CLP/vehicle (*n* = 6), CLP/α-Galcer (*n* = 5). CD1d^KO^: Sham/vehicle (*n* = 3), Sham/α-Galcer (*n* = 3), CLP/vehicle (*n* = 7), CLP/α-Galcer (*n* = 7). (D, E, and G) The expression levels of Ki67, CD25, TGF-β, and IL-10 in Treg cells from spleen and liver were examined using corresponding antibodies. Data are representative of two independent experiments. Sham/vehicle (*n* = 3), Sham/α-Galcer (*n* = 3), CLP/vehicle (*n* = 6), CLP/α-Galcer (*n* = 6). (F) The relative mRNA expression levels of IL-2 in total splenocytes of each group were measured after 24 h of sepsis induction. The results are pooled from three independent experiments. Sham/vehicle (*n* = 6), Sham/α-Galcer (*n* = 7), CLP/vehicle (*n* = 7), CLP/α-Galcer (*n* = 10). (H) The WT mice were transferred with or without 4–5 × 10^5^ Treg cells. The next day, 2 μg α-Galcer was injected followed by CLP. The survival rates of septic mice were assessed over a 150-h monitoring period. No Treg transfer group *n* = 9, Treg transfer group *n* = 7. Each dot represents a mouse sample. Data are representative of two independent experiments. The data are presented as the means ± SEM. Statistical significance was determined using one-way ANOVA with Tukey tests for multiple-group comparisons; survival curve comparison was analyzed by log rank test. ∗*p* < 0.05, ∗∗*p* < 0.01, ∗∗∗*p* < 0.001, ∗∗∗∗*p* < 0.0001.

In post-sepsis, a long-term immunosuppression is partially associated with expanded Treg cells. A recent study has demonstrated that the heightened production of IFN-γ, driven by iNKT cells, can exacerbate susceptibility to secondary infections.^11^ In light of this, we sought to investigate whether iNKT cells could enhance the differentiation of Treg cells upon α-Galcer stimulation. Contrary to our expectations, α-Galcer significantly reduced the expansion of Treg cells in post-sepsis (Figure 2A). Studies have shown that an increased number of Treg cells are followed by the onset of severe sepsis or septic shock, such as 24 h after CLP.^22^ To this end, we examined whether α-Galcer treatment decreased Treg differentiation at early stage of sepsis. Nearly 2-folds increase in the percentage of Treg cells was observed in the spleen and liver 48 h following induction of sepsis comparing with sham mice, while α-Galcer treatment also decreased Treg cell formation (Figure 2B). To know whether the reduction in Treg cell proportion during sepsis is attributed to α-Galcer-induced iNKT cell activation, CD1d^KO^ mice were employed. As anticipated, elimination of NKT cells abolished the α-Galcer-mediated decrease in Treg cells during sepsis (Figure 2C). Subsequently, the proliferative capacity of Treg cells was assessed by examining Ki67 expression. Treatment with α-Galcer significantly diminished Ki67 expression in liver Treg cells and mildly reduced its expression in spleen Treg cells (Figure 2D). Another surface marker of Treg cells, CD25 (IL-2 receptor α), which is constitutively expressed and responsible for cell proliferation in response to IL-2 cytokine signaling, was measured. The administration of α-Galcer resulted in a decline in CD25 expression on Treg cells (Figure 2E). Consistently, the mRNA level of IL-2 in total splenocytes was also decreased upon treatment with α-Galcer in septic mice (Figure 2F), indicating that α-Galcer-triggered iNKT cells reduce the number of Treg cells partially by inhibiting their proliferative capacity. To investigate whether α-Galcer stimulation leads to a reduction in IL-2 expression by iNKT cells, which contributes to the impairment of Treg cell proliferation, we examined the levels of IL-2 expression in iNKT cells. We observed that α-Galcer stimulation resulted in a modest decrease in IL-2 expression by hepatic iNKT cells in septic mice compared to vehicle-septic mice (Figure S2A). However, we observed that α-Galcer stimulation did not result in a decrease in splenic iNKT cell production of IL-2; instead, it led to a reduction in IL-2 expression by conventional T cells. Taken together, α-Galcer stimulation induces a decrease in IL-2 expression by hepatic iNKT cells or splenic conventional T cells in septic mice, thereby partially contributing to the impairment of Treg proliferation. The immunosuppressive cytokines transforming growth factor β (TGF-β) and IL-10 are two representative cytokines expressed by Treg cells. The stimulation of iNKT cells by α-Galcer did not affect the expression of TGF-β and IL-10 in spleen Treg cells during sepsis, but it decreased their production in liver Treg cells (Figure 2G). These findings suggest that α-Galcer-triggered iNKT cells inhibit the increase of Treg cells following the onset of sepsis, and this inhibition does not change during the late stage of sepsis. Figure 1A demonstrates that activation of iNKT cells decreases sepsis survival. To determine whether the decrease in survival induced by α-Galcer is attributed to a reduction in Treg cell numbers, the survival ability was evaluated by additional transfer of Treg cells. It was found that transferring additional Treg cells partially rescued sepsis-induced mortality (Figure 2H).

### iNKT cells inhibit Treg cell formation by IFN-γ

**Figure 3:**
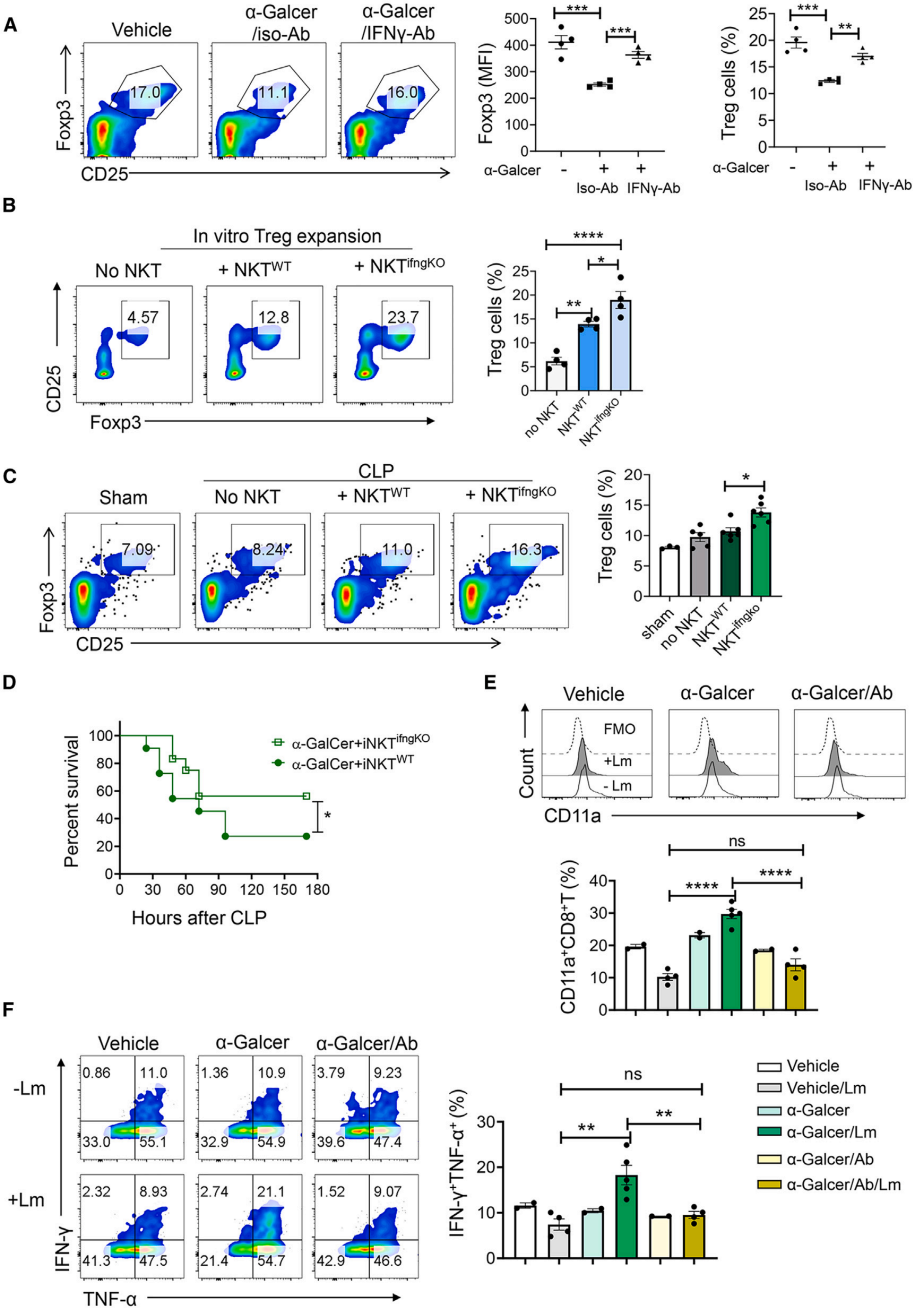
iNKT cells inhibit Treg cell formation via IFN-γ (A) The WT mice were administered with 2 μg α-Galcer or only PBS (vehicle), followed by CLP; 250 μg anti-IFN-γ antibody or its isotype control antibody was then administrated after 1 h of CLP. After 48 h of sepsis induction, Treg cells were examined using FACS analysis. The plots depict the mean fluorescence intensities of Foxp3 in total CD4^+^T cells (left) and the frequencies of Treg cells (right). Each dot represents a mouse sample. Vehicle (*n* = 4), α-Galcer/iso-Ab (*n* = 4), α-Galcer/IFN-γ-Ab (*n* = 4). (B) 1–2 × 10^6^/mL CD8^+^T cell-depleted Ja18^KO^ splenocytes were stimulated for Treg cell differentiation in the presence or absence of 0.25–0.5 × 10^6^/mL iNKT cells that were isolated from WT mice (NKT^WT^) or IFN-γ ^KO^ mice (NKT^ifngKO^). After 4 days of stimulation, frequencies of Treg cells in total cells were determined. *n* = 4 technical replicates. (C) 4–5 × 10^5^ NKT^WT^ or NKT^ifngKO^ cells were transferred to Jα18^KO^ mice. Next day, 2 μg α-Galcer was injected to the mice followed by CLP. The frequencies of Treg cells in the spleen were determined after 48 h of CLP. Each dot represents a mouse sample. Sham (*n* = 3). No NKT (*n* = 5), NKT^WT^ (*n* = 6), NKT^ifngKO^ (*n* = 6). Results are pooled from two independent experiments. (D) 4–5 × 10^5^ NKT^WT^ or NKT^ifngKO^ cells were transferred to Jα18^KO^ mice. Next day, 2 μg α-Galcer was injected to the mice followed by CLP. The survival rates of septic mice were assessed over a 7-day monitoring period. NKT^WT^ transfer group *n* = 11, NKT^ifngKO^ transfer group *n* = 12. The survival curve was determined by combination of two independent experiments. (E and F) The WT mice were administrated with 2 μg α-Galcer or only PBS (vehicle), followed by CLP. After 1 h of CLP, 250 μg anti-IFN-γ antibody was then injected to α-Galcer-pretreated mice. After 14 days of CLP, the survived mice were intravenously injected with or without Lm (2 × 10^7^ colony-forming units [CFUs]/mouse). After 2 days of infection, the expression levels of CD11a (E) and IFN-γ and TNF-α (F) in splenic CD8^+^T cells were examined using corresponding antibodies. Each dot represents a mouse sample. Vehicle (*n* = 2), vehicle/Lm (*n* = 4), α-Galcer (*n* = 2), α-Galcer/Lm (*n* = 5), α-Galcer/Ab (*n* = 2), α-Galcer/Ab/Lm (*n* = 4). The data are presented as the means ± SEM and representative of two (B, E, and F) or three (A) independent experiments. Statistical significance was determined using one-way ANOVA with Tukey tests for multiple-group comparisons. Survival Curve comparison was analyzed by log rank test. ∗*p* < 0.05, ∗∗*p* < 0.01, ∗∗∗*p* < 0.001, ∗∗∗∗*p* < 0.0001.

The results of Figure 1 have shown that α-Galcer elicits robust IFN-γ production and concurrently suppresses IL-4 expression in iNKT cells. Some studies have suggested IFN-γ can suppress Treg cell differentiation.^23^^,^^24^ To know whether upregulated IFN-γ is responsible for inhibition of Treg cell formation in this context, anti-IFN-γ neutralizing antibody was utilized. The expression level of foxp3 in total CD4^+^T cells and the frequency of Treg cells were elevated upon treatment with anti-IFN-γ antibody compared to treatment with an isotype control antibody in septic mice (Figure 3A). To investigate the suppressive role of IFN-γ on Treg cell formation mediated by IFN-γ-producing iNKT cells, CD4^+^T cells were co-cultured with either WT iNKT cells or IFN-γ^KO^ iNKT cells (NKT^ifngKO^) under conditions of IL-2 and TGF-β for promoting Treg cell differentiation. To exclude any contamination of iNKT cells in CD4^+^T cells, Jα18^KO^(without iNKT cells) mice were utilized. The results showed that IFN-γ^KO^ iNKT cells promoted Treg cell differentiation compared to WT NKT cells (Figure 3B). Finally, the role of WT iNKT and IFN-γ^KO^ iNKT cells in the induction of Treg cells was compared *in vivo* through adoptive transfer to Jα18^KO^ mice followed by CLP. Consistently, mice transferred with IFN-γ^KO^ iNKT cells exhibited a higher proportion of Treg cell formation after CLP surgery (Figure 3C). Additionally, the inclusion of iNKT cells in both *in vitro* (Figure 3B) and *in vivo* (Figure 3C) settings also enhances the proportions of Treg cells, indicating the indispensable role of iNKT cells in promoting Treg cell differentiation or survival. Taken together, these data suggest that α-Galcer stimulation induces robust production of IFN-γ by iNKT cells, which inhibits the differentiation of Treg cells during sepsis. We have shown α-Galcer-primed iNKT cells contribute to exacerbation of sepsis (Figures 1A and 1B). α-Galcer stimulation induces high expression of IFN-γ by iNKT cells in sepsis (Figures 1E and 1F). High expression of IFN-γ by iNKT cells results in the inhibition of Treg cell generation (Figures 3A–3C), and transferring additional Treg cells partially rescues sepsis-induced mortality (Figure 2H). Therefore, to examine whether the production of IFN-γ by iNKT cells contributes to exacerbation of sepsis, IFN-γ^KO^ iNKT cells or WT iNKT cells were transferred to Jα18^KO^ mice followed by CLP surgery. IFN-γ^KO^ iNKT cells significantly increased the survival ability of septic mice compared to WT iNKT cells (Figure 3D). Additionally, we observed that α-Galcer stimulation suppressed IL-4 expression by iNKT cells in the septic mice (Figures 1E and 1F). To investigate whether the reduction of IL-4 also contributes to the diminished generation of Tregs following α-Galcer treatment, we additionally administrated IL-4 to α-Galcer-treated mice after CLP surgery and determined the frequencies of Tregs. We found that IL-4 treatment failed to enhance Treg formation in septic mice, indicating that the inhibition of Treg generation was not attributable to α-Galcer-induced impairment of IL-4 production by iNKT cells (Figure S3). Collectively, it indicates that α-Galcer-induced increased susceptibility to sepsis may be due to modulation of Tregs by IFN-γ. To check whether iNKT-derived IFN-γ suppresses Tregs, which would prevent the immunosuppression during post-phase of sepsis, the sepsis-survived mice (14 days of CLP) that are administrated with vehicle or α-Galcer or α-Galcer/IFN-γAb were rechallenged with *Listeria monocytogenes* (Lm). CD11a is a marker associated with the infection-induced naive-to-effector antigen-specific CD8^+^T cell transition *in vivo*.^25^ After 2 days of infection, the frequencies of CD11a^+^ CD8^+^T cells were examined (Figure 3E). In the sepsis-survived mice without Lm infection, the frequencies of CD11a^+^CD8^+^T cells were comparable among the three groups. However, when challenged by Lm, significantly increased frequencies of CD11a^+^ CD8^+^T cells were observed in α-Galcer-pretreated mice, while decreased in vehicle-pretreated mice. Additionally, the level of CD11a^+^ CD8^+^T cells was decreased in the mice co-administered with IFN-γ antibodies and α-Galcer compared to that in only α-Galcer-pretreated mice. Next, we compared the cytokine-expressing capacity among the three groups. The pattern of the frequencies of IFN-γ^+^TNF-α^+^CD8^+^T cells was consistent with that of CD11a^+^CD8^+^T cells (Figure 3F), indicating the higher immune response of CD8^+^T cells in initial α-Galcer-stimulated mice. Therefore, the results suggest that initial α-Galcer stimulation can lead to long-term inhibition of Treg cell generation in sepsis, thereby protecting against immunosuppression in post-sepsis.

### iNKT cells alter the transcriptome of Treg cells in sepsis

IFN-γ has been shown to inhibit Treg cell function by antagonizing the signaling pathways of IL-10 and TGF-β.^26^^,^^27^ Furthermore, Studies have also reported that IFN-γ drives the fragility of Treg cells.^24^ To investigate whether the gene expression profile of Treg cells in α-Galcer treatment septic mice is different from those in vehicle treatment septic mice, the Treg cells were isolated from the spleens of septic mice after α-Galcer or vehicle treatment and their transcriptional landscapes were determined with RNA sequencing.

**Figure 4:**
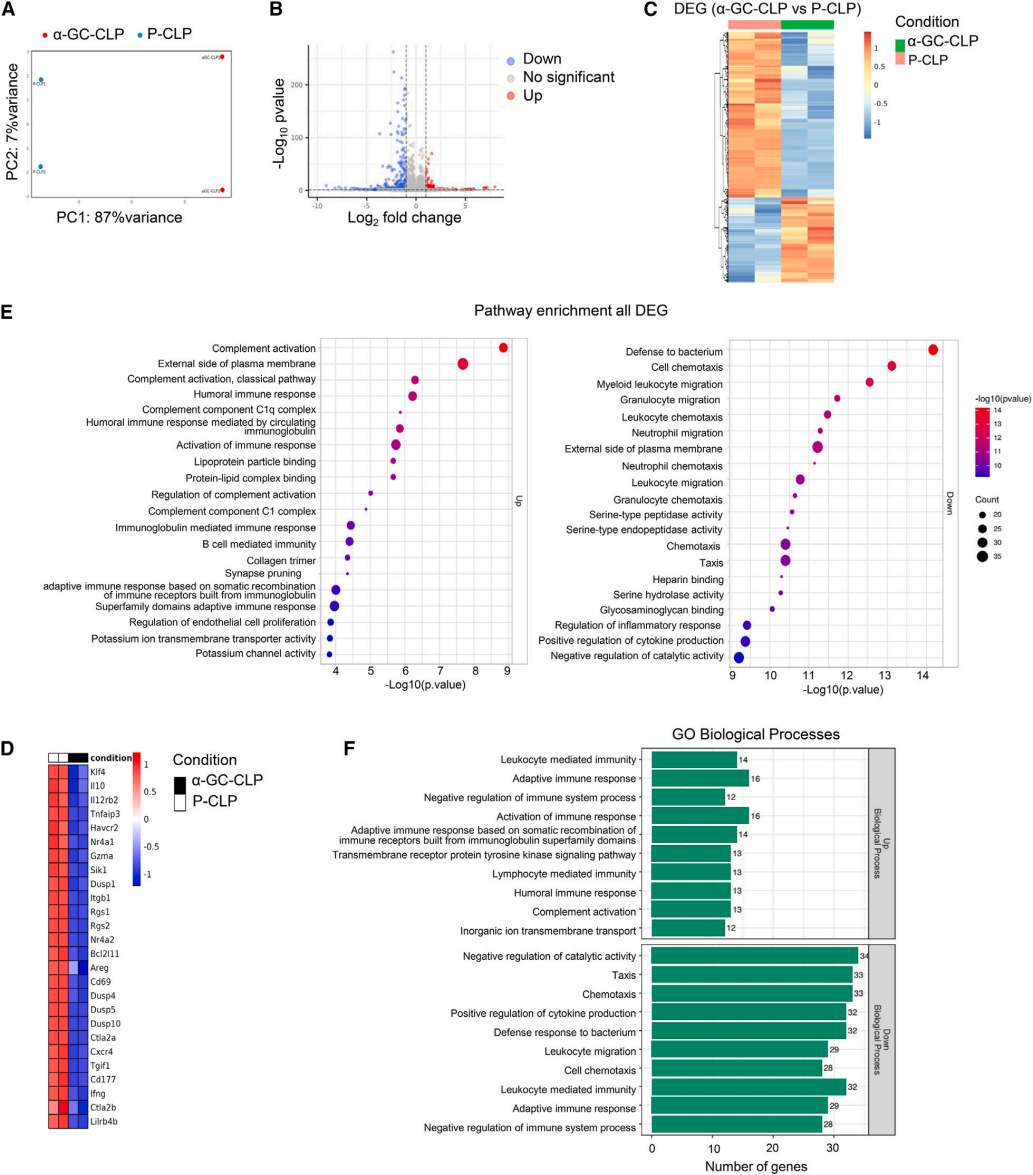
The gene expression profile of α-Galcer-derived Treg cells is different from that of vehicle-derived Treg cells (A) Principal-component analysis demonstrating the clustering differences between conditioned Treg cells. (B) Volcano plot demonstrating 220 genes upregulated and 428 genes downregulated in α-Galcer-derived Tregs relative to vehicle-derived Tregs (Wald test, Bonferroni multiple test correction). (C) Heatmap demonstrating hierarchical clustering of the differentially expressed genes between the two types of Treg cells. (D) Heatmap showing the normalized count values for the Treg cells that are associated with their effector function, functional stability, and differentiation. (E) Graph displaying the pathways enriched among differentially expressed genes. (F) Bar graph highlighting the GO biological processes associated with genes that are upregulated or downregulated in α-Galcer-derived Treg cells.

An unsupervised principal-component analysis (PCA) was performed to study the variability between Treg cells derived from different environments. α-Galcer-derived Treg cells clearly clustered separately from vehicle-derived Treg cells (Figure 4A), indicating that activation of iNKT cells modulates Treg cells with a specific expression pattern compared to conventional Treg cells. There are 220 genes upregulated and 428 genes downregulated in α-Galcer-derived Treg cells compared to vehicle-derived Tregs (Figures 4B and 4C). Pathway analysis identified that the differentially expressed genes were associated with downregulated pathways linked to defense response to bacterium, cell chemotaxis, myeloid leukocyte migration, regulation of inflammatory response, and positive regulation of cytokine production (Figure 4E). Genes that were upregulated in α-Galcer-derived Treg cells were enriched for the following Gene Ontology (GO) biological processes: leukocyte-mediated immunity (*n* = 14), adaptive response (*n* = 16), negative regulation of immune system process (*n* = 12), and lymphocyte-mediated immunity (*n* = 13). But mRNA transcripts that were downregulated in α-Galcer-derived Treg cells were associated with positive regulation of cytokine production (*n* = 32), defense response to bacterium (*n* = 32), leukocyte migration (*n* = 29), cell chemotaxis (*n* = 28), leukocyte-mediated immunity (*n* = 32), adaptive immune response (*n* = 29), and negative regulation of immune system process (*n* = 28) GO biological processes (Figure 4F). Notably, the genes related to suppressive effector function or responsible for stabilization of foxp3 expression, such as il10, gzma, cd69, ctla, lilr4b, and dusp4, were downregulated in α-Galcer-derived Tregs (Figure 4D). These results indicated that effector functions of α-Galcer-derived Treg cells might be impaired.

### IFN-γ-producing iNKT cells inhibit Treg cell formation via the IFN-γ-Nr4a1 axis

**Figure 5:**
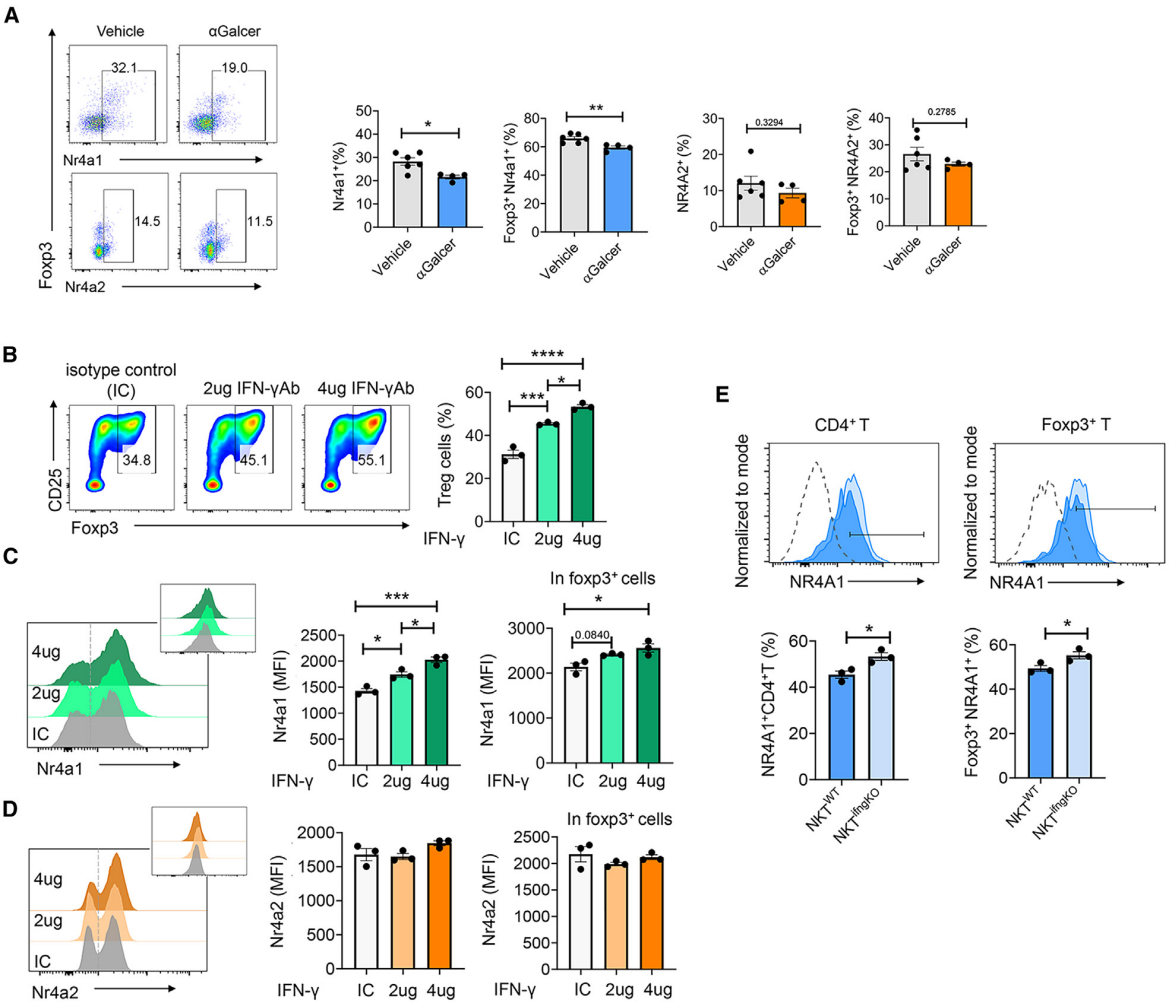
iNKT cells inhibit Treg cell formation via the IFN-γ-NR4A1 axis (A) The WT mice were administrated with 2 μg α-Galcer or only PBS (vehicle), followed by CLP. After 48 h of sepsis induction, the expression levels of Nr4a1 and Nr4a2 were detected in total CD4^+^T cells and Treg cells via intracellular staining. Vehicle (*n* = 6), α-Galcer (*n* = 4). (B–D) Naive CD4^+^T cells were isolated from WT mice and stimulated with anti-CD3/CD28 in the presence of IL-2 and TGF-β to induce Treg cell differentiation. Additionally, IFN-γ neutralizing antibodies or their isotype control antibodies were treated in the culture. After 48 h stimulation, the frequencies of Treg cells were examined (B). The expression levels of Nr4a1 (C) and Nr4a2 (D) were assessed in both total CD4^+^T cells and Treg cells. The large histogram represents the expression of Nr4a1 or Nr4a2 in CD4^+^T cells, while the small histogram represents their expression in Treg cells. *n* = 3 technical replicates. (E) 1–2 × 10^6^/mL CD8^+^T cell-depleted Jα18^KO^ splenocytes were stimulated with anti-CD3/CD28 in the presence of IL-2 and TGF-β to induce Treg cell differentiation in the presence of NKT cells that were isolated from WT mice (NKT^WT^) or IFN-γ KO(NKT^ifngko^) mice. 0.1 μg/mL α-Galcer was treated in the culture medium for iNKT cell activation. After 48 h of stimulation, the frequencies of Nr4a1^+^CD4^+^T cell and Nr4a1^+^Foxp3^+^T cell were examined. *n* = 3 technical replicates. The experiments were conducted two (A and E) or three (B–D) times yielding consistent results. Statistical significance was determined using one-way ANOVA with Tukey tests for multiple-group comparisons (B–D) and unpaired two-tailed Student’s t test (A and E). Data are shown as the mean ± SEM. ∗*p* < 0.05, ∗∗*p* < 0.01, ∗∗∗*p* < 0.001, ∗∗∗∗*p* < 0.0001.

The Nr4a family of nuclear orphan receptors, including Nr4a1, Nr4a2, and Nr4a3,^28^ exhibit ligand-independent transcriptional activity due to their unique conformation resembling that of ligand-bound transcription factors. Therefore, the transcriptional activity of Nr4a receptors is dependent on their expression levels, interaction partners, and posttranscriptional modification.^29^ A series of recent studies revealed that the Nr4a family is crucial for the differentiation and maintenance of Treg cells.^30^^,^^31^ Foxp3 can be induced by Nr4a factors; deletion of Nr4a factors results in a lack of Treg cell differentiation. Nr4a factors also help maintain the stability of Treg cell lineage via activation of the Treg cell-associated genes and repression of the Th-effector genes.^32^ Thus, to identify the potential mechanisms underlying how α-Galcer-activated iNKT cells impair Treg cell differentiation and effector function, Nr4a1 and Nr4a2 that are significantly downregulated in mRNA level in α-Galcer-derived Tregs were focused (Figure 4D). First, we determined whether activation of iNKT cells impaired the expression level of Nr4a1 and Nr4a2 in total CD4^+^T cells and Treg cells following CLP surgery. A significant reduction in Nr4a1 expression was observed in CD4^+^ T cells and Treg cells of α-Galcer-treated septic mice, while there was no notable decrease in the level of Nr4a2 expression. (Figure 5A). We have demonstrated that the differentiation of Treg cells is suppressed by IFN-γ secreted by α-Galcer-stimulated iNKT cells (Figure 4), which prompted us to investigate whether neutralizing IFN-γ could reduce the expression levels of Nr4a factors. Naive CD4^+^T cells were isolated and subjected to forced Treg cell differentiation *in vitro*. As expected, treatment with neutralizing antibodies against IFN-γ enhanced Treg cell differentiation (Figure 5B) and also promoted the expression of Nr4a1 in both CD4^+^T cells and Treg cells (Figure 5C). However, neutralization of IFN-γ did not increase Nr4a2 expression in either CD4^+^T cells or Treg cells (Figure 5D). To further determine whether the IFN-γ produced by α-Galcer-activated iNKT cells suppresses Nr4a1 expression, CD8^+^T cell-depleted splenocytes of Ja18^KO^ mice were stimulated for Treg cell differentiation in the presence WT NKT or IFN-γ^KO^ NKT cells. After 3 days of stimulation, increased levels of Nr4a1 expression were observed in CD4^+^T cells, and a mild increase of Nr4a1 was also detected in Treg cells (Figure 5E). These results indicated that the activated iNKT cells suppress Treg cell formation through decrease of Nr4a1 expression during sepsis.

Overall, our data determined that α-Galcer-primed iNKT cells skewing toward Th1 type not only inhibit the differentiation of Treg cells but also impair effector function of Treg cells during sepsis. Notably, the inhibition of Treg cell formation persists for long term in the post-sepsis phase. Mechanistically, we reveal that iNKT cells mediate the suppression of Treg cell formation through the IFN-γ-Nr4a1 axis. Therefore, our findings provide a novel role of iNKT cells in the pathogenesis of sepsis. The modulation of iNKT cells represents a promising therapeutic target for ameliorating their adverse long-term consequences.

## Discussion

Sepsis is the leading cause of mortality resulting from severe infection worldwide. Regrettably, there are currently no specific therapeutic modalities available for septic patients other than infection control and organ function support.^2^^,^^33^ Although patients with sepsis typically exhibit a profoundly dysregulated immune system, characterized by the state from excessive inflammation to long-term immunoparalysis, the intricate immunity of sepsis remains largely unknown. Thus, a better understanding of the immunopathogenesis of this syndrome will contribute to the development of innovative therapies.

NKT cells, as the primary initiators of the immune response, have been demonstrated to play critical roles in the pathogenesis of sepsis. However, their mechanism remains largely unknown. Our data demonstrate that IFN-γ-producing iNKT cells play a pathogenic role in early sepsis by suppressing Treg cell differentiation, and adoptive transfer of Treg cells can reduce sepsis-induced mortality. Deficiency in IFN-γ production by iNKT cells significantly enhances Treg cell differentiation, partially dependent on the transcription factor Nr4a1. Transcriptome analysis of Treg cells suggested that iNKT cells also impaired the effector functions of Treg cells. Thus, our findings suggest that activation of iNKT cells leading to IFN-γ production can exacerbate early sepsis by inhibiting Treg cell differentiation and effector function through an IFN-γ-Nr4a1 axis. However, Treg cells are a double-edged sword in sepsis. They regulate the immune response to restrict excessive inflammation and minimize damage to host tissues during early sepsis. Conversely, an increased frequency of Treg cells is associated with long-term sepsis-induced immunosuppression.^13^ In fact, our findings suggest that iNKT cell-mediated suppression of Treg cell differentiation persists over long term, indicating its potential for mitigating sepsis-induced immunosuppression. This mechanism accounts for, at least in part, the role of IFN-γ-producing iNKT cells in the progress of sepsis.

Consistent with our findings, several studies demonstrated that IFN-γ has detrimental effects on hosts with sepsis.^10^^,^^11^^,^^34^^,^^35^ Treatment with anti-IFN-γ antibodies significantly decreases mortality in the CLP-induced sepsis model, whereas the administration of recombinant IFN-γ significantly enhances it.^36^ One mechanism suggested IFN-γ-producing iNKT cells exacerbate early sepsis by enhancing a complement C5a mediated by another innate cell neutrophils.^10^ A recent study focused on post-sepsis has suggested that iNKT cells induce downstream production of IFN-γ, which in turn increases the risk of secondary infection in the host.^11^ These two separate studies highlight the critical roles played by iNKT cells and IFN-γ in both early and late sepsis, providing partial support for our own findings.

The transcriptome analysis was compared between the two conditioned Treg cells, and it revealed a decrease in genes associated with effector function (such as il10, gzma, ctla, and lilr4b) in iNKT cell-modulated Treg cells. Dual-specificity phosphatase 4 (DUSP4), responsible for stabilizing the expression of Foxp3^37^ and enhancing the expression of Treg transcriptional signature,^38^ was also downregulated in iNKT cell-modulated Treg cells. These findings suggest that iNKT cells producing IFN-γ not only inhibit the generation of Treg cells but also compromise their effector function and stability during sepsis. This observation supports the notion that IFN-γ inhibits Treg cell differentiation and functions within tumor microenvironments.^23^^,^^24^^,^^39^ Furthermore, activation of iNKT cells can also induce the downstream cells, such as NK cells and type 1 lymphoid cells (ILC1s), and production of IFN-γ,^40^ which further augments the impact of iNKT cells on the differentiation and function of Treg cells.

Among the “Treg signature genes,” the Nr4a factors were focused since they have been addressed responsible for Treg cell differentiation. We observed the protein level of Nr4a1 factor was decreased in both CD4^+^T cells and Treg cells after treatment with α-Galcer. For neutralization of IFN-γ under the condition of Treg cell differentiation from naive CD4^+^T cells, we observed that the expression of Nr4a1 was elevated in both CD4 and Treg cells. IFN-γ is considered a Th1-type cytokine due to its ability to induce T-bet, a transcription factor that controls the genetic program of Th1 cell differentiation in naive CD4^+^ T cells.^41^ One proposed molecular mechanism suggests that Nr4a factors promote the differentiation of Treg cells by suppressing T-bet expression,^42^ which further supports our findings on the impairment of Treg cell differentiation by IFN-γ signals through downregulation of Nr4a1 factors.

In conclusion, our data provide a novel role of iNKT cells in the regulation of Treg cell differentiation during sepsis. The activated iNKT cells predominantly skew toward Th1 type, exerting inhibitory effects on Treg cell differentiation and impairing effector function through the downregulation of Nr4a1 expression, which persists throughout the late stage of sepsis. Regarding to the double-edged sword role of Treg cells in the two stages of sepsis, it provides a new potential target on modulation of iNKT cells in reversing the immunosuppression of late stage of sepsis.

### Limitations of the study

A limitation of the current study is that it was conducted exclusively on murine models, leaving unanswered the question of whether the role of iNKT cells in human sepsis aligns with their function in mice. Additionally, this study does not investigate the molecular mechanism underlying how IFN-γ inhibits Nr4a1 expression in Treg cells. Further investigations should be directed toward elucidating this aspect.

## Resource availability

### Lead contact

Further information and requests for resources and reagents should be directed to and will be fulfilled by the lead contact, Dr. Yingyu Qin (yyqin@seu.edu.cn).

### Materials availability

This study did not generate new unique reagents.

### Data and code availability

• The bulk RNA sequencing dataset generated in this study has been deposited at the China National GeneBank DataBase (CNGBdb) and is publicly available as of the date of publication. Accession numbers are listed in the key resources table.
• This paper does not report original code.
• Any additional information required to reanalyze the data reported in this paper is available from the lead contact upon request.

## Acknowledgments

This work was supported by grants from the 10.13039/501100001809National Natural Science Foundation of China (32100712), 10.13039/100017962Jiangsu Provincial Health Commission Key project (K2023005), and 10.13039/501100012226Fundamental Research Funds for the Central Universities (2242022R40060).

## Author contributions

Y.Qin. and R.C. designed research studies. Y. Qin, Y. Qian, and S.L. conducted the experiments. Y. Qin, Y. Qian, and S.L. acquired and analyzed data. Y.Qin. and S.L. wrote the manuscript. Y.Qin. and R.C. edited and finalized the manuscript. All authors contributed to the article and approved the submitted version.

## Declaration of interests

The authors declare no competing interests.

## STAR★Methods

### Key resources table

**Table:** 

REAGENT or RESOURCE	SOURCE	IDENTIFIER
**Antibodies**		

APC/Cyanine7 anti-mouse TCR β chain	BioLegend	Cat# 109220 RRID: AB_893626
FITC anti-mouse CD4	BioLegend	Cat# 100406 RRID: AB_312690
Pacific Blue™ anti-mouse CD4	BioLegend	Cat# 100531 RRID: AB_493374
PE/Dazzle™ 594 anti-mouse CD25	BioLegend	Cat# 101920 RRID: AB_2721701
FOXP3 Monoclonal Antibody (FJK-16s), APC	Thermo Fisher Scientific	Cat# 17-5773-82; RRID: AB_469457
Brilliant Violet 421™ anti-mouse Ki-67	BioLegend	Cat# 652411 RRID: AB_2562663
PE anti-mouse LAP (TGF-β1)	BioLegend	Cat# 141404 RRID: AB_10730610
PE/Cyanine7 anti-mouse IL-10	BioLegend	Cat# 505026 RRID: AB_11149682
FITC anti-mouse NK-1.1	BioLegend	Cat# 156508 RRID: AB_2876526
PerCP/Cyanine5.5 anti-mouse CD3	BioLegend	Cat# 100218 RRID: AB_1595492
APC anti-mouse IFN-γ	BioLegend	Cat# 505810 RRID: AB_315403
PE anti-mouse IL-4	BioLegend	Cat# 504104 RRID: AB_315317
PerCP/Cyanine5.5 anti-mouse CD127 (IL-7Rα)	BioLegend	Cat# 135022 RRID: AB_1937274
PE/Cyanine7 anti-mouse IL-2	BioLegend	Cat# 503832 RRID: AB_2561749
Nur77 Monoclonal Antibody (12.14), PE	Thermo Fisher Scientific	Cat# 12-5965-82 RRID: AB_1257209
Nr4a2-FITC	Biorbyt	Cat# orb2128524
CD8α-Biotin	BioLegend	Cat# 100704 RRID: AB_312742
InVivoMAb anti-mouse IFNγ	BioXCell	Cat# BE0054 RRID: AB_1107692
InVivoMAb rat IgG1 isotype control	BioXCell	Cat# BE0088 RRID: AB_1107775
anti-mouse CD3ε	BioLegend	Cat# 100340 RRID: AB_2800555
InVivoMAb anti-mouse CD28	BioXCell	Cat# BE0015-1 RRID: AB_1107624

**Bacterial and virus strains**		

*Listeria monocytogenes*	BeNa Culture Collection	BNCC336877

**Chemicals, peptides, and recombinant proteins**		

Percoll	Cytiva	Cat# 17089101
TRIzol™ Reagent	Thermo Fisher Scientific	Cat# 15596018
RPMI 1640 Medium	Gibco	Cat# 11875-093
FBS	Ozfan	Cat# FBSKM0502
Penicilllin-streptomycin	Sigma Aldrich	Cat# P4333
Pentobarbital sodium	Sigma Aldrich	Cat# P3761 CAS# 57-33-0
Mouse IL-2 Recombinant Protein	PeproTech®	Cat# 1212-12-5UG
Mouse TGF-β1 Recombinant Protein	UA BiOSCIENCE	Cat# UA040172
Mouse IL-4 Recombinant Protein	MedChemExpress	Cat# HY-P70653
α-Galactosylceramide (KRN7000)	Avanti Research	Cat# 867000

**Critical commercial assays**		

Foxp3 Transcription Factor Staining Buffer Kit	Thermo Fisher Scientific	Cat# 00-5523-00
Cell Activation Cocktail (with Brefeldin A)	BioLegend	Cat# 423304
LIVE/DEAD Fixable Blue viability	Thermo Fisher Scientific	Cat# L23105
EasySep™ Mouse CD4^+^ T cell Isolation Kit	STEMCELL	Catalog # 19852
EasySep™ Mouse CD4^+^CD25^+^ Regulatory T cell Isolation Kit II	STEMCELL	Catalog # 18783
NK1.1+ iNKT Cell Isolation Kit, mouse	Miltenyi Biotec	Catalog # 130-096-513
Naive CD4^+^ T cell Isolation Kit, mouse	Miltenyi Biotec	Catalog # 130-104-453
ChamQ SYBR qPCR Master Mix	Vazyme	Catalog #Q331-02

**Deposited data**		

Bulk RNA-sequencing data	This paper	CNGBdb: CNP0005968; https://db.cngb.org/

**Experimental models: organisms/strains**		

C57BL/6JNifdc	GemPharmatech, Jiangsu, China	N/A
B6(Cg)-Traj18tm1.1Kro/J (Jα18^KO^)	Xinzhi Wang et al.^43^	N/A
B6.129S6-Del(3Cd1d2-Cd1d1)1Sbp/J (CD1d^KO^)	The Jackson Laboratory	RRID: IMSR_JAX:00888
C57BL/6JGpt-Ifngem3Cd10241/Gpt (IFNγ^KO^)	GemPharmatech, Jiangsu, China	Strain #: T012669

**Oligonucleotides**		

IL-2: Forward: 5′-TGAGCAGGATGGAGAATTACAGG -3′ and Reverse: 5′-GTCCAAGTTCATCTTCTAGGCAC -3'.	This paper	N/A
GAPDH: Forward: 5′- CATCACTGCCACCCAGAAGACTG-3’; Reverse: 5′-ATGCCAGTGAGCTTCCCGTTCAG -3’.	This paper	N/A

**Software and algorithms**		

GraphPad Prism v8	GraphPad Software	https://www.graphpad.com/
FlowJo™ version 10.8.1	BD	https://www.flowjo.com/solutions/flowjo/downloads

### Experimental model and study participant details

#### Animals

Jα18 knockout (Jα18^KO^) mice were kindly provided by Pro. Xinzhi Wang^43^ (China Pharmaceutical University, Nanjing, China). CD1d^KO^ mice were purchased from Jackson laboratories and kindly provided by Pro. Zhigang Lei (Nanjing Medical University, Nanjing, China). The IFN-γ ^KO^ mice and C57BL/6 mice were purchased from Model Animal Research Center of Nanjing University (GemPharmatech, Jiangsu, China). All mice were kept in specific pathogen free animal facilities of Southeast University and selected to ensure age (6–9 weeks) and sex matching in experiments. All animal experiments were approved by institutional guidelines established by the Committee of Ethics on Animal Experiments of Southeast University under protocol number 20210205002.

#### Bacterial stains

We utilized *Listeria monocytogenes* (BNCC336877) procured from BeNa Culture Collection for secondary infection. Listeria monocytogenes was cultured in Brain Heart Infusion (BHI) medium.

### Method details

#### Sepsis induction by cecal ligation and puncture

To induce sepsis in mice, a small incision was made along the abdominal midline of mice that were anesthetized with 1% pentobarbital sodium (1 mg/kg). The cecum was exposed approximately 1 cm from the end of cecal tip and ligated using 3-0 silk. A single puncture was then made the ligated cecum using a 26-gauge needle. The mice in the sham surgery group underwent a surgical procedure that did not involve ligation and puncture. For α-Galcer-mediated iNKT cell activation, 2 μg α-galcer in 100 μl PBS or only PBS was intraperitoneal injected before surgical. For neutralization of endogenous IFN-γ *in vivo*, 250 μg anti- IFN-γ antibody or an isotype control IgG1K was intraperitoneally injected after 1 h of CLP. For IL-4 administration, after 1 h and the following day of performing CLP, IL-4 was injected at a dose of 1 μg each time.

#### Treg cell isolation and RNA-seq analysis

The CD4^+^ T cells were isolated from splenocytes of septic mice treated with α-galcer or PBS (two days after CLP performance) using the negative CD4^+^T cell isolation kit, following the manufacturer’s instructions. Subsequently, the Treg cell population, characterized by CD25^high^CD127^low^ markers, was sorted using BD FACSAria Fusion Flow Cytometers. To ensure an adequate number of enriched Treg cells, Treg cells were collected from three mice. The isolated Treg cells with a purity about 90% were subjected to TRIzol treatment, and total RNA was extracted according to the manufacturer’s protocol.

The libraries were sequenced on a llumina Novaseq 6000 platform. The reads were mapped to the mouse genome using HISAT2. Then the FPKM of each gene was calculated and the read counts of each gene were obtained by HTSeq-count. The biological duplication of samples was assessed by performing PCA analysis in R (v 3.2.0). Differential expression analysis was performed using the DESeq25. A threshold of Q value <0.05 and fold change >2 or foldchange <0.5 was set to identify significantly differential expression genes (DEGs). Hierarchical cluster analysis of DEGs was performed using R (v 3.2.0) to demonstrate the expression pattern of genes in different groups and samples.

#### Real-time quantitative PCR

The spleens were isolated from septic mice 24 h after CLP, followed by TRIzol treatment and subsequent RNA extraction. Quantitative real-time PCR (qPCR) was performed with the use of SYBR Green, on a QuantStudio 3 Real-Time PCR System (Thermo fisher). All reactions were run in triplicates.

#### Flow cytometric analysis

Splenocytes and liver lymphocytes were analyzed with below antibodies: TCRβ-APC.Cy7 (H57-597), CD4-FITC (GK1.5), CD25-PE/Dazzle594/(3C7), Foxp3-APC (FJK-16s), Ki67-BV421 (16A8), TGF-β-PE (TW7-16B4), IL-10-PE.Cy7 (JES5-16E3), NK1.1-FITC (S17016D), CD3-Percp5.5 (17A2), IFN-γ-APC (XMG1.2), IL-4-PE (11B11), IL-2-PE.Cy7, Nr4a1-PE (12.14), Nr4a2-FITC (polyclonal). LIVE/DEAD Fixable Blue viability (Invitrogen) were incubated with other indicated antibodies during surface staining to exclude dead cells. For intracellular staining, the cells were first stained with the indicated surface antibodies. Subsequently, they were fixed and permeabilized using a Foxp3 Transcription Factor Staining Buffer set following the manufacturer’s protocols. For intracellular cytokine analysis, the cells were stimulated with Cell Activation Cocktail (with PMA, ionomycin, and Brefeldin A) for 5–6 h at 37°C, 5%CO_2_ prior to staining.

Data acquisition was performed on a five-laser BD LSRFortessa Cell Analyzer Flow Cytometer using appropriate filter sets and compensation controls. Gate assignment was based on suitable control populations.

#### Adoptive transfer experiments

Treg cells were isolated from splenocytes of wild type C57BL/6 mice via the CD4^+^CD25^+^ Regulatory T cell Isolation Kit II (STEMCELL) according to the manufacturer’s instructions. The wild type mice were intravenously injected with 4–5×10^5^ Treg cells. The next day, 2 μg α-Galcer was intraperitoneally injected, followed by CLP. For NKT cell isolation and transfer, the liver lymphocytes were enriched using gradient centrifugation (40% Percoll, 600 × g, 15 min). Subsequently, NKT cells were isolated from splenocytes and liver lymphocytes of wild type C57BL/6 mice or IFN-γ ^KO^ mice by NK1.1^+^ iNKT Cell Isolation Kit. Ja18^KO^ mice were infused with 4–5×10^5^ NKT cells isolated from either WT or IFN-γ^KO^ mice. The next day, the NKT cell-transferred mice were injected with 2 μg α-Galcer subsequent to CLP.

#### Infection of Listeria monocytogenes (Lm)

The WT mice were injected with 2 μg α-Galcer or only PBS (vehicle), followed by CLP. After 1 h of CLP, 250 μg anti-IFN-γ antibody was then intraperitoneally injected to α-Galcer-pretreated mice. After 14 days of CLP, the survived mice were intravenously injected with or without Lm (2 × 10^7^CFU/mouse).

#### Polarization of Treg cells *in vitro*

1–2×10^6^/mL CD8^+^T cell-depleted Ja18^KO^ splenocytes (negatively sorted by CD8α-biotin/anti-biotin-magnetic beads) were stimulated with plate-bond anti-CD3/CD28 in the presence or absence of 0.25–0.5×10^6^/mL NKT cells (from WT or IFN-γ ^KO^ mice) with additional 0.1 μg/mL α-Galcer treatment, after 2 or 3 days of stimulation, the frequencies of Treg cells and Nr4a1expression were determined using FACS analysis.

Naive CD4^+^T cell were isolated using Naive CD4^+^ T cell Isolation Kit (Miltenyi Biotec) according to the manufacturer’s instructions and the purified 4×10^6^/mL naive CD4^+^T cells were stimulated with plate-bond anti-CD3/CD28 in the presence of IL-2 (10 ng/mL) and TGF-β (10 ng/mL), a determined amount (2 μg, 4 μg) of anti-IFN-γ antibodies or isotype control IgG1K were additionally treated for neutralizing endogenous IFN-γ. After 48h stimulation the expression levels of Nr4a1and Nr4a2 in expanded CD4^+^T cells and Foxp3^+^Treg cells were examined using FACS analysis.

### Quantification and statistical analysis

Statistical data analysis was conducted using GraphPad Prism (version 8). The differences between two datasets were determined using unpaired, two-tailed student t tests. One-way or two-way ANOVA was used for multi-group comparisons. Survival Curve comparison was analyzed by log rank test. Significance was considered at *p* < 0.05 (∗), *p* < 0.01 (∗∗), *p* < 0.001 (∗∗∗), and *p* < 0.0001(∗∗∗∗) levels. The data are expressed as the mean±the standard error of the mean (SEM), and representative results from two or three independent repeats with similar results are shown.
