# Supplementary material for: A double-edged sword role of IFN-γ-producing iNKT cells in sepsis: Persistent suppression of Treg cell formation in an Nr4a1-dependent manner
Source: iScience. 2024 Nov 23;27(12):111462. doi: 10.1016/j.isci.2024.111462 (PMC11667017; doi:10.1016/j.isci.2024.111462)
Supplement: Document S1. Figures S1–S3 [file mmc1.pdf]

iScience, Volume *27*

Supplemental information

A double-edged sword role of IFN-γ-producing iNKT cells in sepsis: Persistent suppression of Treg cell formation in an Nr4a1-dependent manner

Yingyu Qin, Yilin Qian, Shengqiu Liu, and Rong Chen

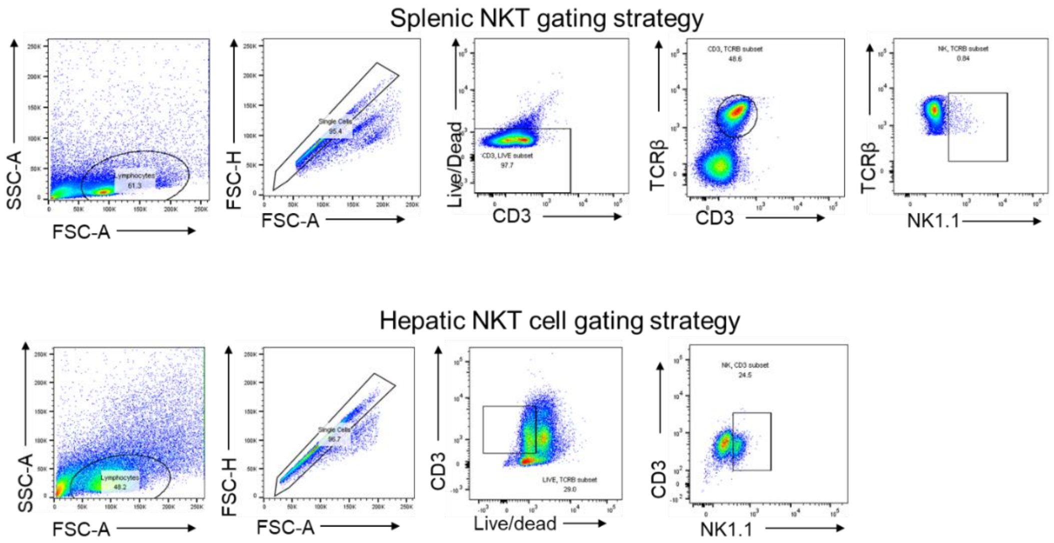

Figure S1. Gating strategy of NKT cells, related to Figure 1.

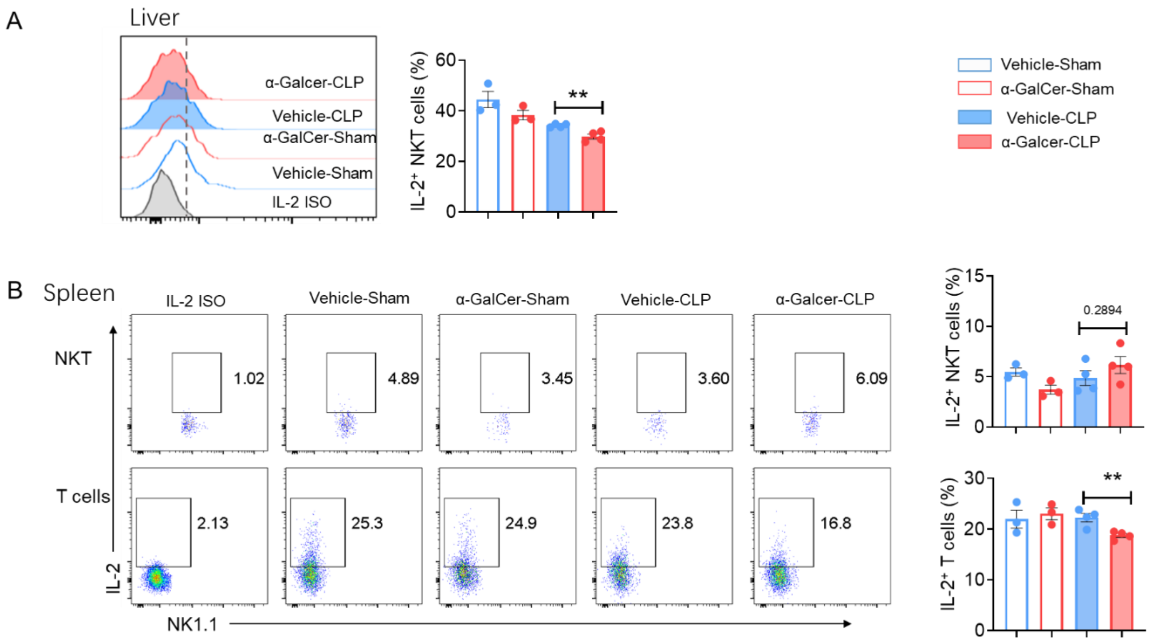

Figure S2. Detection of IL-2 expression by NKT or conventional T cells in sham or CLP mice, related to Figure 2.

A-B. The WT mice were intraperitoneally injected with 2 μg α-Galcer in PBS or only PBS (vehicle), followed by CLP or sham surgery. After 12 hours of sepsis induction, the expression levels of IL-2 in hepatic NKT cells (A) or spleen NKT cells (B, upper panel) or spleen T cells (B, lower panel) were analyzed using intracellular FACS analysis. Each dot represents a mouse sample. Vehicle-sham (n=3), α-Galcer-sham (n=3), Vehicle-CLP (n=4), α-Galcer-CLP (n=4). Data are representative of two independent experiments. Statistical significance was determined using one-way ANOVA with Tukey tests for multiple-group comparisons. Data are shown as the mean ± SEM. **P < 0.01

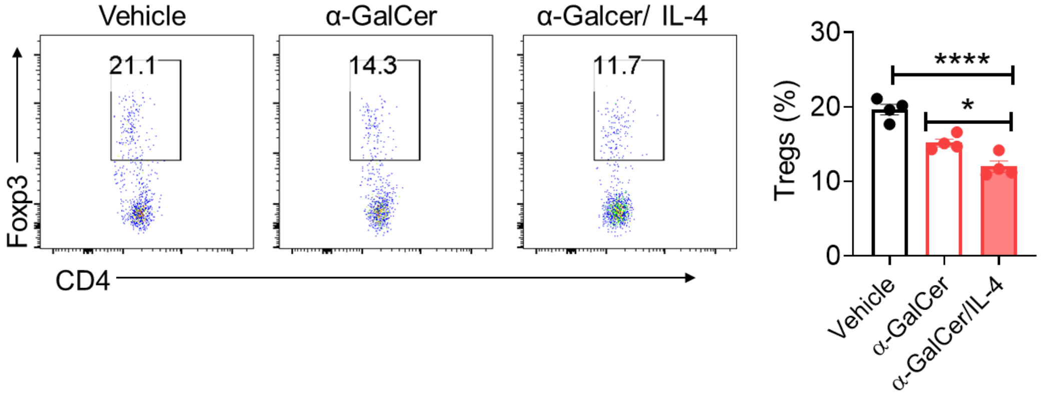

Figure S3. Examination of the impact of exogenous IL-4 administration on Tregs, Related Figure 3.

The WT mice were administered with 2 μg α-Galcer or only PBS (vehicle), followed by CLP. After 1 hour and the following day of CLP, 1 μg IL-4 was injected each time. After 48 hours of sepsis induction, Treg cells were examined using FACS analysis. Each dot represents a mouse sample. Data are representative of two independent experiments. Statistical significance was determined using one-way ANOVA with Tukey tests for multiple-group comparisons. *$P < 0.05$, ****$P < 0.0001$.
